# Local Control Rates of Metastatic Renal Cell Carcinoma (RCC) to Thoracic, Abdominal, and Soft Tissue Lesions Using Stereotactic Body Radiotherapy (SBRT)

**DOI:** 10.1186/s13014-015-0528-z

**Published:** 2015-10-28

**Authors:** Basel Altoos, Arya Amini, Muthanna Yacoub, Maria T. Bourlon, Elizabeth E. Kessler, Thomas W. Flaig, Christine M. Fisher, Brian D. Kavanagh, Elaine T. Lam, Sana D. Karam

**Affiliations:** Department of Radiation Oncology, University of Colorado School of Medicine, 1665 Aurora Court, Room 1032, Aurora, CO 80045 USA; University of Iowa Carver College of Medicine, Iowa City, Iowa USA; Medical Oncology Department, Instituto Nacional de Ciencias Médicas y Nutrición Salvador Zubirán, Mexico City, Mexico; Department of Medicine, Division of Medical Oncology, University of Colorado School of Medicine, Aurora, Colorado USA

**Keywords:** Renal cell carcinoma, Local control, SBRT, Conventional fractionation

## Abstract

**Background and purpose:**

We report the radiographic response rate of SBRT compared to conventional fractionated radiotherapy (CF-EBRT) for thoracic, abdominal, skin and soft tissue RCC lesions treated at our institution.

**Material and methods:**

Fifty three lesions where included in the study (36 SBRT, 17 CF-EBRT), treated from 2004 to 2014 at our institution. We included patients that had thoracic, skin & soft tissue (SST), and abdominal metastases of histologically confirmed RCC. The most common SBRT fractionation was 50 Gy in 5 fractions.

**Results:**

The median time of follow-up was 16 months (range 3–97 months). Median BED was 216.67 (range 66.67–460.0) for SBRT, and 60 (range 46.67–100.83) for CF-EBRT. Median radiographic local control rates at 12, 24, and 36 months were 100, 93.41, and 93.41 % for lesions treated with SBRT versus 62.02, 35.27 and 35.27 % for those treated with CF-EBRT (*p* < 0.001). Predictive factors for radiographic local control under univariate analysis included BED ≥ 100 Gy (HR, 0.048; 95 % CI, 0.006–0.382; *p =* 0.005), dose per fraction ≥ 9 Gy (HR, 0.631; 95 % CI, 0.429–0.931; *p =* 0.021), and gender (HR, 0.254; 95 % CI, 0.066–0.978; *p* = 0.048). Under multivariate analysis, there were no significant predictors for local control. Toxicity rates were low and equivalent in both groups, with no grade 4 or 5 side effects reported.

**Conclusions:**

SBRT is safe and effective for the treatment of RCC metastases to thoracic, abdominal and integumentary soft tissues. Radiographic response rates were greater and more durable using SBRT compared to CF-EBRT. Further prospective trials are needed to evaluate efficacy and safety of SBRT for RCC metastases.

## Background

The global annual incidence of renal cell carcinoma (RCC) is nearly 209,000 cases, killing over 100,000 people each year [5]. The majority of these patients present with isolated disease, however, about 30 % of these patients recur after treatment either locally or, more frequently, distantly and about the same percentage present with metastatic spread at the time of diagnosis [18]. In the setting of metastatic disease, RCC most commonly spreads to the lung, accounting for approximately 60 % of metastatic cases. Hepatic involvement occurs in about 20 % of cases. Prognosis is generally favorable for patients with non-metastatic disease however the two-year survival in patients with widespread disease is much worse (10–20 %) [18].

As patients with RCC continue to live longer given the rapid advances seen in systemic treatment for RCC with targeted agents (eg, tyrosine kinase inhibitors (TKIs), vascular endothelial growth factor (VEGF) inhibitors, and mTOR inhibitors), localized treatment of enlarging or anatomically problematic masses is becoming increasingly utilized to further lengthen and improve quality of life [4, 6, 11]. In patients with oligo-metastatic disease, surgical resection has been employed with promising results. For instance, Loizzi et al. demonstrated improved overall survival, four-year survival of 50 %, after surgically resecting easily accessible RCC lung lesions [9].

In addition to surgical therapy, traditionally fractionated radiotherapy has been used to treat metastatic disease despite the traditional thinking of RCC being “radioresistant”. However, over the past decade, increased use of hypofractionation with stereotactic body radiotherapy (SBRT) has allowed clinicians to accurately target lesions and deliver more ablative doses. Efficacy of SBRT has been demonstrated in multiple metastatic sites, with local control rates above 90 % [13, 17, 18]. Bextracranial stereotactic radiotherapyextracranial stereotactic radiotherapysingle-institution studies demonstrating high local control rates using SBRT, we sought to directly compare the radiographic outcomes of RCC lesions between traditional or conventional fractionation (CF-EBRT) and SBRT in our institution to enhance the dataset analyzing the radiosensitivity of RCC. Additionally, the goal of this study was to further define the most effective radiation delivery by estimating a minimum dose, either fraction size or biologic effective dose (BED) cutoff, needed to achieve long term local control.

## Materials and methods

Patients were retrospectively identified by searching an institutional database of patients treated with radiation for RCC at the University of Colorado Cancer Center between December 2005 and May 2014. This study was approved by the University of Colorado Institutional Review Board. We included patients that had pathological confirmation of primary RCC and radiographic evidence of metastatic lesions. All patients included had a minimum follow up of 3 months post radiation therapy. Sites of metastasis were grouped into thorax, skin & soft tissue (SST), and abdomen.

Patients were treated with SBRT using intensity modulated radiotherapy (IMRT) planning. All patients treated with SBRT underwent treatment simulation prior. Simulation scans were CT based, with 3 mm slice thickness. In the more recent years, lesions with tumor motion underwent 4D imaging with abdominal compression. Patients were supine and immobilized with a vac-loc or alphacrade. IV contrast use was at the discretion of the treating physician. For treatment planning, the gross tumor volume (GTV) was considered equal to the clinical target volume (CTV). An internal target volume (ITV) was generated for lesions with motion who underwent 4D imaging, which was then expanded to the planned tumor volume (PTV). The PTV expansion depended on the treatment site and physician discretion. CF-EBRT were treated with simple 2D planning, 3D, and at times intensity modulated radiotherapy (IMRT) if there were nearby critical organs at risk nearing maximum dose tolerance. All SBRT cases underwent daily image guidance (IGRT) during treatment.

Patients were evaluated for radiographic response after treatment. Radiographic complete response was defined as: no evidence of disease in the treatment volume by interpretation of available Fludeoxyglucose-Positron emission tomography/computed tomography (FDG-PET/CT), Magnetic Resonance Imaging (MRI), or computed tomography (CT) scans 3 months after SBRT. Stable disease was defined as absence of marked change or increase in the treated lesion. Partial response was defined as not meeting the criteria for complete response or stable disease. Patients were labeled as radiographic responders if they had a complete response, partial response and/or stable disease after radiation. Treated metastasis control was defined as the time from the last day of radiation treatment to failure at the treated site or last follow-up in living patients without evidence of recurrence or progression. Use of the Response Evaluation Criteria in Solid Tumors (RECIST) was not used due to difficulty assessing parenchymal changes common after SBRT, especially to sites treated to 20 Gy or higher. Instead failure at the treated site was scored as lesions that experienced a complete, partial or stable response after therapy; all other lesions that had an increase in standardized uptake value (SUV) on PET or expansion of a solid mass compared to prior CT-based imaging. All scans were verified by a certified radiologist.

Univariate survival analysis was performed with the log-rank test, with Cox proportional hazards regression used to estimate hazard ratios (HR). Multivariate Cox regression analysis was performed to evaluate the association between clinical factors and LRC with a significance level of *p* < 0.05. The independent variables considered were total dose, number of fractions, dose per fraction, biologic effective dose (BED), site (thorax vs other), histology (clear cell vs other), GTV, PTV, gender, age, time from initial diagnoses to end of radiation therapy, smoking status, extensive disease (as defined by greater than five metastatic lesions), and systemic therapy pre- and post-radiation. CTCAE version 4.0 was used for scoring toxicity. Patients were censored at the time of death and lost to follow up.

## Results

### Patient and treatment characteristics

In total, 34 patients were selected, with 53 treated lesions; 36 (68 %) lesions were treated with SBRT and 17 (32 %) with conventional fractionation (CF-EBRT). The study cohort was followed for a median time of 16 months (range 3–97 months) and the median time for first follow-up was similar in both groups. The majority of patients were male (59 %) with clear cell histology (91 %). The most common SBRT fractionation was 50 Gy given in 5 fractions (10 Gy per fraction); the most common CF-EBRT treatment was 40 Gy in 10 fractions (4 Gy per fraction); 47 % (*N* = 8) of CF-EBRT were treated with palliative intent (20 Gy in 5 fractions/30 Gy in 10 fractions) (Table 1). The majority of lesions treated were located in the thorax [lung parenchyma (N = 23), hilar and mediastinal lymph nodes (*N* = 12), and pericardium (*N* = 1)]. Eight lesions were located in skin/soft tissue [skin/soft tissue extremity (*N* = 5), parotid (*N* = 1), gingiva (*N* = 1), and tongue (*N* = 1)], and nine lesions treated were within the abdomen [abdominal lymph nodes (*N* = 3), kidney (*N* = 3), pancreas (*N* = 2), and liver (*N* = 1)]. Median number of lesions treated per patient was 1 (range 1–3). There was no difference between median tumor size between the two groups (SBRT - 21 mm (4–87) and CF-EBRT - 23 mm (15–57)) after *t*-test comparison (*p* = 0.357). Median BED was 216.67 (range 66.67–460.0) and 60 (range 46.67–100.83) for SBRT and CF-EBRT respectively. The majority of patients were on systemic therapy before (72 %) and after radiation (57 %) (Table 2).

**Table 1:** Patient and clinical characteristics

Variable	Value
Total Number of Patients	34
Age	
Median (range)	68 (27–84)
Sex	
Male	20 (59 %)
Female	14 (41 %)
KPS	
Median (range)	90 (60–100)
Histology	
Clear Cell	48 (91 %)
Papillary	4 (7 %)
Not Otherwise Specified	1 (2 %)
Treatment Patterns	
Treated with SBRT	36 (68 %)
25 Gy/1 fx	2
30 Gy/2 fx	1
54 Gy/4 fx	1
24–60 Gy/3 fx	15
25–50 Gy/5 fx	14
40 Gy/8 fx	1
50 Gy/10 fx	2
Treated with CF-EBRT	17 (32 %)
20 Gy/5 fx	5
32 Gy/8 fx	1
30–40 Gy/10 fx	8
45 Gy/13 fx	1
45 Gy/15 fx	1
55 Gy/22 fx	1

*KPS* karnofsky performance status, *SBRT* stereotactic body radiotherapy, CF*-EBRT* conventional fractionated external beam radiotherapy

**Table 2:** Treatment characteristics between SBRT and CF-EBRT

Variable	SBRT (*n* = 36)	CF-EBRT (*n* = 17)
Location, n (%)		
Thorax	27 (75 %)	9 (53 %)
Skin & Soft Tissue	3 (8.3 %)	5 (29 %)
Abdomen	6 (16.7 %)	3 (18 %)
Median Dose Per Fraction (range, Gy)	10 (5–25)	4 (2.5–4)
Median Tumor Size (range, mm)*	21 (4–87)	23 (15–57)
Gross Tumor Volume (GTV) (range, cm^3^)	5.14 (0.93–184.07)	-
Planned Tumor Volume (PTV) (range, cm^3^)	20.01 (6.00–295.51)	-
Biological Effective Dose (BED) (range)	216.67 (66.67–460)	60.00 (46.67–100.83)
Systemic Therapy Prior to Radiation, n (%)		
Yes	26 (72 %)	12 (71 %)
No	10 (28 %)	5 (29 %)
Systemic Therapy After Radiation, n (%)		
Yes	20 (55 %)	10 (59 %)
No	16 (45 %)	7 (41 %)

*GTV* gross tumor volume, *PTV* planned tumor volume, *BED* biologic effective dose

*As determined from the greatest tumor dimension by pre-treatment radiographic imaging. No difference was found between the two groups via *t*-test comparison (*p* = 0.357)

### Response rates

**Fig. 1:**
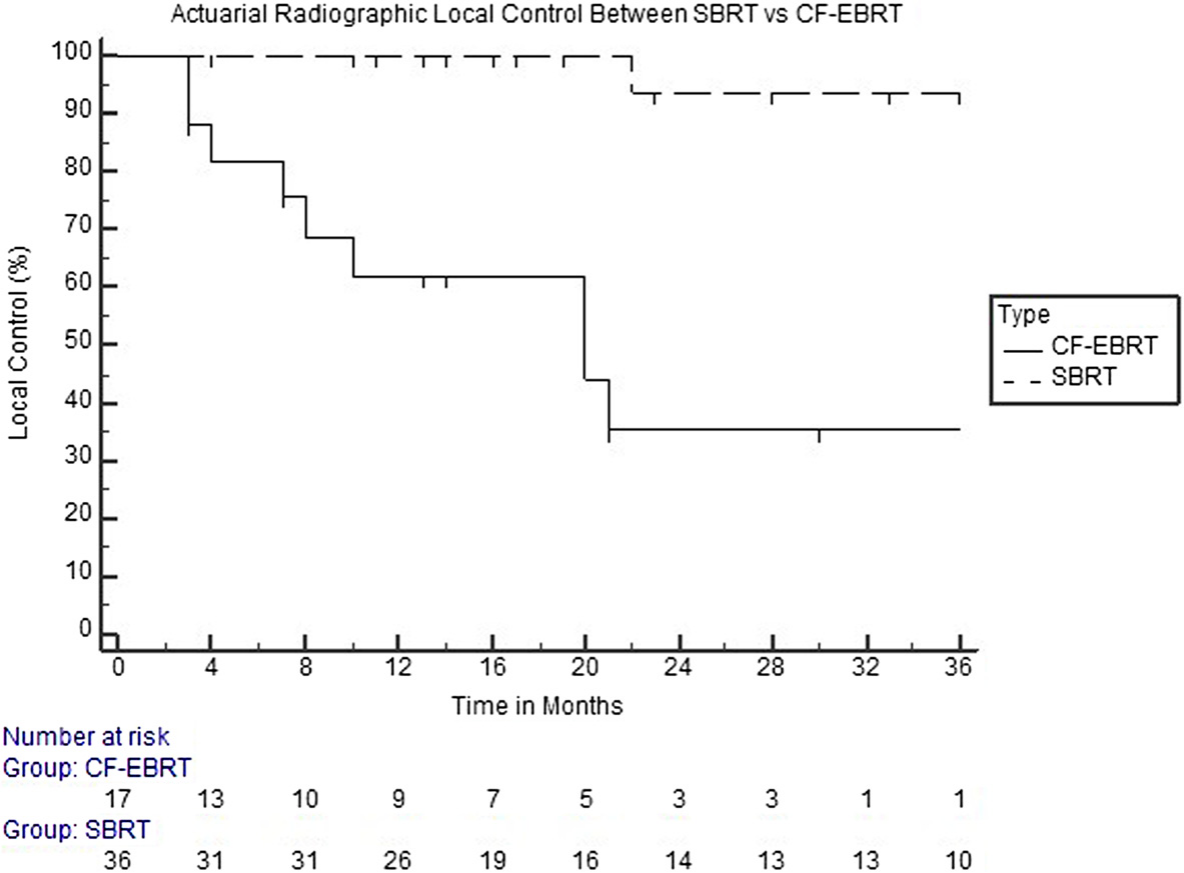
Kaplan-Meier curve demonstrating actuarial radiographic control rates between SBRT and CF-EBRT

**Fig. 2:**
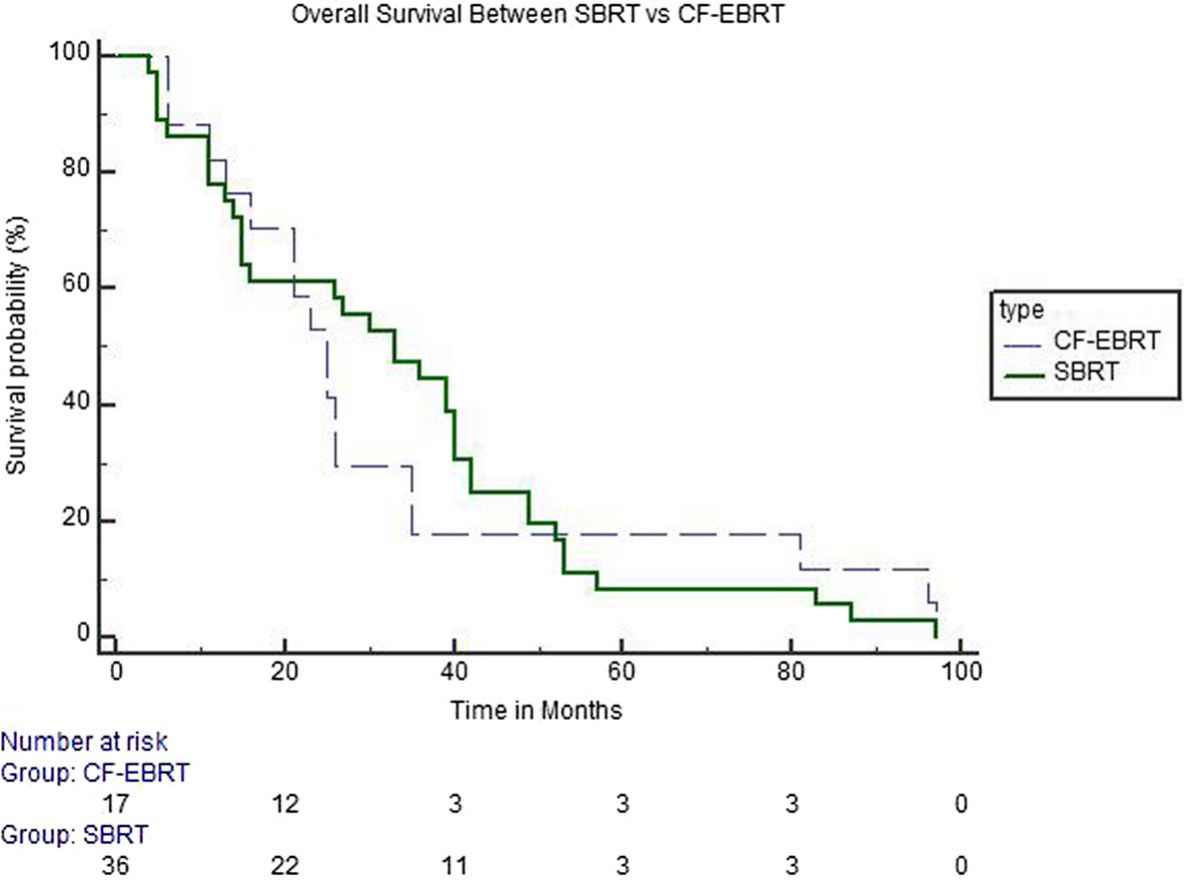
Kaplan-Meier curve demonstrating Overall Survival Rates between SBRT and CF-EBRT

For all patients combined, radiation therapy provided a median 12 and 24 months treated metastasis control rate of 87.45 and 72.16 %, respectively. The median 12 and 24 months treated metastasis control rate in SBRT treated lesions were 100 and 93.41 % versus 62.02 and 35.27 % in the CF-EBRT (Fig. 1). Radiographic local control rates were significantly different (*p* < 0.001). There was no difference in overall survival between the two groups (*p* = 0.831) (Fig. 2).

### Predictors for local control

When combining all patients included in the study (SBRT and CF-EBRT), predictive factors for radiographic local control under univariate analysis included BED ≥ 100 Gy (HR, 0.048; 95 % CI, 0.006–0.382; *p =* 0.005) and a dose per fraction ≥ 9 Gy (HR, 0.631; 95 % CI, 0.429–0.931; *p =* 0.021). Another predictor included gender (HR, 0.254; 95 % CI, 0.066–0.978; *p* = 0.048). Non-significant predictive factors for radiographic local control included age, lesion location, total dose, smoking, and systemic therapy before or after treatment. GTV and PTV size were non-significant for SBRT patients (*p =* 0.429). All significant predictors under univariate analysis did not maintain significance under multivariate analysis (Tables 3, 4 and 5). Toxicity rates were low and equivalent in both groups, with no grade 4 or 5 side effects reported (Table 6).

**Table 3:** Univariate and multivariate analysis for radiographic predictors of Local Control (LC) for all patients

	Univariate	Multivariate	
Variable	*P*-value	HR (95 % CI)	*P*-value
Total Dose (Gy)	0.001*	0.999 (0.998 to 1.001)	0.209
Number of Fractions	0.189	1.004 (0.849 to 1.185)	0.966
Dose per Fractions (≥9 Gy)	0.021	0.726 (0.469 to 1.124)	0.153
BED (100 Gy)	0.005*	0.264 (0.022 to 3.119)	0.293
Site (Thorax vs Other)	0.185	1.941 (0.515 to 7.323)	0.329
Time from Primary Diagnosis	0.318	0.996 (0.980 to 1.012)	0.663
Male Gender	0.048	0.481 (0.120 to 1.915)	0.301
Age	0.610	0.990 (0.916 to 1.071)	0.816
Smoker	0.123	0.276 (0.058 to 1.311)	0.107
Greater than 5 Metastatic Lesions	0.808	0.674 (0.170 to 2.673)	0.577
Pre-Radiation Systemic Therapy	0.517	2.299 (0.605 to 8.724)	0.223
Post-Radiation Systemic Therapy	0.332	0.201 (0.038 to 1.037)	0.056

*BED* biologic effective dose, *HR* hazard ratio, *CI* confidence interval

*-Statistically significant values

**Table 4:** Univariate and multivariate analysis for radiographic predictors of Local Control (LC) for SBRT patients

	Univariate	Multivariate	
Variable	*P*-value	HR (95 % CI)	*P*-value
Total Dose (Gy)	0.772	0.999 (0.997 to 1.001)	0.608
Number of Fractions	0.789	1.086 (0.528 to 2.233)	0.822
Dose per Fractions (≥9 Gy)	0.474	0.835 (0.528 to 1.319)	0.442
BED (100 Gy)	0.534	0.988 (0.957 to 1.019)	0.461
Site (Thorax vs Other)	0.961	0.948 (0.915 to 1.012)	0.957
GTV	0.429	0.958 (0.921 to 1.091)	0.967
PTV	0.429	-	-
Time from Primary Diagnosis	0.491	1.224 (0.645 to 2.320)	0.537
Age	0.701	1.063 (0.885 to 1.277)	0.513
Smoker	0.965	-	-
Greater than 5 Metastatic Lesions	0.963	-	-
Pre-Radiation Systemic Therapy	0.959	-	-
Post-Radiation Systemic Therapy	0.961	-	-

*BED* biologic effective dose, *GTV* gross tumor volume, *HR* hazard ration, *CI* confidence interval

**Table 5:** Univariate and multivariate analysis for radiographic predictors of Local Control (LC) for CF-EBRT patients

	Univariate	Multivariate	
Variable	*P*-value	HR (95 % CI)	*P*-value
Total Dose (Gy)	0.025*	0.994 (0.986 to 1.003)	0.216
Number of Fractions	0.114	0.872 (0.698 to 1.090)	0.232
Dose per Fractions (≥9 Gy)	0.950	0.729 (0.195 to 2.725)	0.640
BED (100 Gy)	0.017*	3.94 (0.028 to 2.345)	0.325
Site (Thorax vs Other)	0.867	1.579 (0.404 to 6.173)	0.513
Time from Primary Diagnosis	0.714	0.987 (0.961 to 1.015)	0.386
Male Gender	0.653	1.003 (0.244 to 4.128)	0.995
Age	0.560	0.953 (0.860 to 1.056)	0.366
Smoker	0.172	-	-
Greater than 5 Metastatic Lesions	0.933	0.886 (0.216 to 3.623)	0.867
Pre-Radiation Systemic Therapy	0.536	2.340 (0.586 to 9.345)	0.231
Post-Radiation Systemic Therapy	0.417	0.206 (0.035 to 1.242)	0.086

*BED* biologic effective dose, *HR* hazard ration, *CI* confidence interval

*-Statistically significant values

**Table 6:** Toxicities (*N* = 31)

Toxicity	Grade 1	Grade 2	Grade 3
Esophagitis	2	0	0
Edema	2	0	0
Mucositis	3	2	1
Pneumonitis	1	2	0
Fatigue	15	1	0
Dermatitis	2	0	0
Emesis	1	1	0
Nausea	3	1	0
Hiccups	0	1	0
SOB	1	0	0
Pain	0	1	0

No Grade 4 or 5 toxicities to report

## Discussion

As assessment of radiation therapy’s effectiveness in RCC is dependent on the BED administered and this work provides an important comparison between SBRT and EBRT in this regard. Presently, the majority of SBRT data for RCC tumors focuses on osseous metastatic sites, with less data on non-bony locations. In the era of SBRT, our findings in addition to other single institutional studies, have demonstrated long-term radiographic local control rates for RCC lesions with the use of SBRT instead of CF-EBRT. The data presented here is an update of prior data published at our institution, which had included 25 lesions (in 13 RCC patients) treated with SBRT; the results here, which includes more lesions with longer follow up, confirms our prior findings that aggressive SBRT can result in good local control rates. Additional institutions have also demonstrated similar results [12, 18]. Wersall and colleagues published their results and demonstrated local control rates above 90 % in their series of RCC treated sites including lung, renal bed, lymph nodes, and adrenal gland; 30 % of patients had complete regression of lesions within 3–36 months, 60 % with partial volume reduction or no change after a median follow-up of 37 months, 2 % had progression, and 8 % were non-evaluable due to early death or atelectasis 18]. Svedman et al. demonstrated very similar findings with a 98 % local control rate. Complete response in their cohort was observed in 21 % of the patients and 58 % of the patients had a partial volume reduction or local stable disease after a median follow-up of 52 months [16]. These findings are similar to our results, with local control rates of 90 % or higher. Taken together, these single institutional studies suggest that SBRT may be more effective at controlling RCC lesions, which were once thought to be radioresistant with the use of lower dose, conventional therapy.

The results presented here also illustrate predictors for local response included BED ≥ 100 and fraction size ≥ 9 Gy. These findings are again supported by earlier results published at our institution evaluating the use of SBRT and doses needed to establish effective control rates for metastatic melanoma and RCC [1, 15]. Stinauer and colleagues included 25 RCC lesions treated with 40–50 Gy in 5 fractions or 42–60 Gy in 3 fractions and had a local control rate of 88 % at 18 months [15]. Predictors of in-field local control included BED > 100 Gy and fraction size > 11 Gy, comparable to our findings. BED has been confirmed to be a strong predictive factor in many other studies, including several Radiation Therapy Oncology Group (RTOG) clinical trials evaluating local control rates with SBRT [10, 13, 17]. Radiobiologically, the higher dose per fraction with SBRT-based treatments has been shown to provide improved local control over standard fractionation. As the survival and proliferation of tumor cells are directly dependent on the blood supply, SBRT has been shown to have a direct effect on tumor vasculature. Hypoxia can increase the expression of vascular endothelial growth factors, which is associated with higher grade tumors and metastatic disease [3]. High-dose single fractionation at 10 Gy or higher has been shown to cause severe vascular damage in human tumor xenografts or animal tumors [2, 8]. SBRT, coupled with targeted agents including vascular endothelial growth factor (VEGF) inhibitors and TKIs, may have both a synergistic local effect in addition to systemic effect and is currently being studied. Additionally, the vascular injury and ensuing chaotic intratumor environment such as hypoxic, acidic and nutritionally-deprived environment caused by high-dose fraction SBRT, may significantly hinder the repair of radiation damage [14]. RCC has several hypothesized mechanisms for radiation resistance with EBRT. One may be a mutation or silencing of the von Hippel-Lindau (VHL) gene which is present in over 50 % of cases and is thought to lead in the accumulation of hypoxic-inducible factor 1-alpha (HIF1A), which then creates angiogenesis leading to further tumor growth and potential disease spread [7]. As described, the mechanism in which SBRT can cause endothelial apoptosis with single high dose treatments may help overcome this pathway.

Currently, the role of systemic therapy in RCC continues to improve with more targeted therapies, providing patients with improved survival and quality of life. Because of this, SBRT is positioned to play a valuable role in providing not only high local control rates but prolonged duration of symptomatic control from months to years. This in turn could translate into both symptomatic relief for the patients and improved survival, especially in those with oligo-progressive disease. In addition, new agents are constantly being developed that may have a synergistic effect with SBRT and will need to be evaluated in clinical trials.

Our study is limited by its retrospective nature, short-term follow up which may have underestimated disease recurrence rates and long term toxicity, and variability in treatment (specifically, fractionation size and total dosage). A common weakness in these studies is the inherent selection bias that may exist as patients who are treated with SBRT may have had less systemic burden, lower comorbidities, and better overall performance status. However, as our endpoints focuses on local radiographic control, it is unlikely these factors contribute significantly to our findings. Further, this study includes data over a long period of time, where technical and systemic treatment approaches have vastly changed. Additionally, due to the relative small numbers of lesions included in this study, subset analysis of each group was difficult. The majority of our patients who underwent SBRT were asked to hold their systemic therapies such as TKIs the day prior to starting SBRT and resumed the day after completion; future studies would need to evaluate toxicity of combining TKIs and other targeted agents with SBRT. Lastly, long term follow up of how these treatments influence the overall survival of patients with RCC is unknown and worthy of prospective follow up.

To conclude, based on our results and emerging data from other investigators, a difference in efficacy between SBRT and CF-EBRT is emerging in the treatment of RCC. Future prospective studies are needed to evaluate efficacy and toxicity of SBRT in the setting of oligometastatic disease for RCC, especially in the setting of newer immune-modulating therapies. With the current published single institutional studies available, SBRT appears to significantly improve local control rates and symptom relief for metastatic RCC to the bone and should be considered for these patients.
